# The antinociceptive effect of electroacupuncture at different depths of acupoints and under the needling surface

**DOI:** 10.1186/1749-8546-7-3

**Published:** 2012-02-27

**Authors:** Marcelo L Silva, Josie RT Silva, Wiliam A Prado

**Affiliations:** 1 Department of Pharmacology, Faculty of Medicine of Ribeirão Preto-USP, Avenue Bandeirantes 3900, CEP 14049-900 Ribeirão Preto, SP, Brazil

## Abstract

**Background:**

The stimulation of acupoints along the meridians, but not the non-acupoints outside of the meridians, produces analgesia. Although the acupoint is defined at the body surface, the exact location of the acupoints is not known. This study aims to examine whether the intensity and duration of the analgesic effect of electroacupuncture (EA) at the *Zusanli *(ST36) and *Sanynjiao *acupoints (SP6) change according to the depth of the stimulation.

**Methods:**

Ninety-six male Wistar rats classified as responders were arbitrarily allocated into 16 groups of six rats each. Six groups received EA with uninsulated acupuncture needles (type I) or needles that were immersed in varnish and had the varnish circularly peeled 0.2 mm from the tip (type II), 0.2 mm at 3 mm (type III) or 5 mm (type IV) from the tip, or 0.2 mm at 5 and 1 mm from the tip (type V), or EA sham for 20 min. Five groups received injection of formalin into the acupoint bilaterally at 5 mm or 1 mm deep into ST36, 5 mm below ST36 but inserting the needle at 45° to the skin surface, or 5 mm deep into non-acupoints. The remaining groups received intraplantar injection of saline, 1% or 2.5% formalin. The analgesic effects were measured by the rat tail-flick test.

**Results:**

The bilateral stimulation of ST36 and SP6 by uninsulated or insulated needles produced analgesia in the rat tail-flick test. The stronger and longer lasting effects occurred after EA with the types I and V needles, or injection of formalin 5 mm deep into ST36. The remaining needles produced weaker and shorter lasting effects. Slow analgesic effect also occurred after formalin injection at 1 mm or 5 mm below ST36 by inserting the needle at 45° to the skin surface.

**Conclusion:**

The experimental results suggest that the efficacy of the EA stimulation depends on the spatial distribution of the current density under the needling surface rather than only the acupoint or the depth of needling.

## Background

Acupuncture is a Chinese medicine treatment through inserting needles into specific sites known as acupoints on the body's surface [1]. Acupuncture is particularly useful for pain relief, however, little is understood about its biological basis [2]. The acupoints can be stimulated by different methods, including manual needling, the application of electrical stimulation or heat to the acupuncture needle, or by applying pressure or laser-generated light to the acupoint. According to Chinese medicine theory, there is a network of meridian channel inside the human body connecting the acupoints on the skin and deeper tissues [3]. The stimulation of acupoints along the meridians produces analgesia [4]. The evidence for the physical existence of acupoints and meridians includes a low electrical impedance at acupoints [5,6] and the flow of radioisotopes along the meridians [7,8]. The effects of acupuncture have been related to the impulses starting in muscles or other deep tissues [9], and association of the acupoints to the connective tissue [10].

The infiltration of deep nerves with lignocaine around an acupoint impairs the acupuncture-related analgesic response [11], indicating that neural innervations are involved in the acupuncture response [12,13]. However, the sensory mechanisms of acupuncture and electroacupuncture (EA) that initiate the afferent nerve discharge are not fully understood [14,15]. The winding of tissues around the needle caused by manual acupuncture and the subsequent activation of sensory mechanoreceptors and nociceptors have been described [14]. The subcutaneous muscle layer of the rat skin is described as being distinctively different from the surrounding skeletal muscle fibers [15].

The needles used for manual acupuncture or EA are typically made of uninsulated silver or stainless steel. During the passage of an electric current through a metallic needle, its electric field strength is highly concentrated at the needle tip and decays rapidly with increasing distance from the tip [16]. Although the acupoint is defined on the body surface, the exact point of stimulation is not known. The analgesic effect of acupuncture was replicated by the direct injection of 2-chloro-N(6)-cyclopentyladenosine (CCPA), an adenosine A1 receptor agonist into the acupoint [17]. The injection of bee venom, formalin, or complete Freund's adjuvant (CFA) into the *Zusanli *acupoint (ST36) significantly inhibited intraplantar bee venom-induced persistent spontaneous nociception and mechanical hyperalgesia in rats, indicating that the acupoints are also sensitive to chemical acupuncture [18].

This article evaluated the hypothesis that acupoints have an anatomical correspondence with connective tissue planes. This study used the rat tail-flick test to examine whether the intensity and duration of the analgesic effect of EA at ST36 and the *Sanynjiao *acupoints (SP6) or the injection of formalin into ST36 change according to the depth of the stimulation. The measurability of the electric current density throughout the entire needle track was also necessary for observation of the effect.

## Methods

### Animals

The experiments were conducted on male Wistar rats (140-160 g; age 5-6 weeks) from the main animal house of the University of São Paulo (USP; Campus of Ribeirão Preto, Brazil). The experiments were approved by the Commission of Ethics in Animal Research, Faculty of Medicine of Ribeirão Preto, University of São Paulo (Number 078/2011). The guidelines of the Committee for Research and Ethical Issues of International Association for the Study of Pain [19] were followed throughout the experiments. Each rat was used only once.

### Tail-flick test

Each animal was placed in a ventilated tube with the tail laid across a wire coil maintained at room temperature (21-25°C). The coil temperature was raised by the passage of electric current, and the latency for the tail withdrawal reflex was measured. Heat was applied to a portion of the ventral surface of the tail between 4 and 6 cm from the tip. Each trial was terminated after 6 s to minimize the possibility of skin damage. The tail-flick latency (TFL) was measured at 5 min intervals until a stable baseline was obtained in three consecutive trials. Only rats showing a stable baseline latency after up to six trials were used in subsequent experiments.

### Selection of animals

The analgesic effect of acupuncture was subject to individual differences that were reported in human [20] and rodents [21], classifiable as responders or non-responders [22]. Each animal used in the experiments was preliminarily tested for responder classification by the tail-flick test after a 10 min period of EA application to ST36 and SP6 at a frequency of 2 Hz. The responder test was conducted on the rats lightly anesthetized with isoflurane (in oxygen flow through a loose-fitting, cone-shaped mask; 2% for induction and 0.5% for maintenance). Tang *et al*. [23] classified responder rats as low or high responders for an increase of at least 30% or 60%, respectively, in the nociceptive threshold after EA. In order to minimize the possible misinterpretation of the reduction in the TFL, we considered an increase of at least 90% in the nociceptive threshold as the minimum to classify positive responders to the antinociceptive effect of EA. The TFL of a responder was more than 5.5 s 5 or 10 min after beginning the EA. The responder rats were taken for further experiments.

### Electroacupuncture

**Figure 1:**
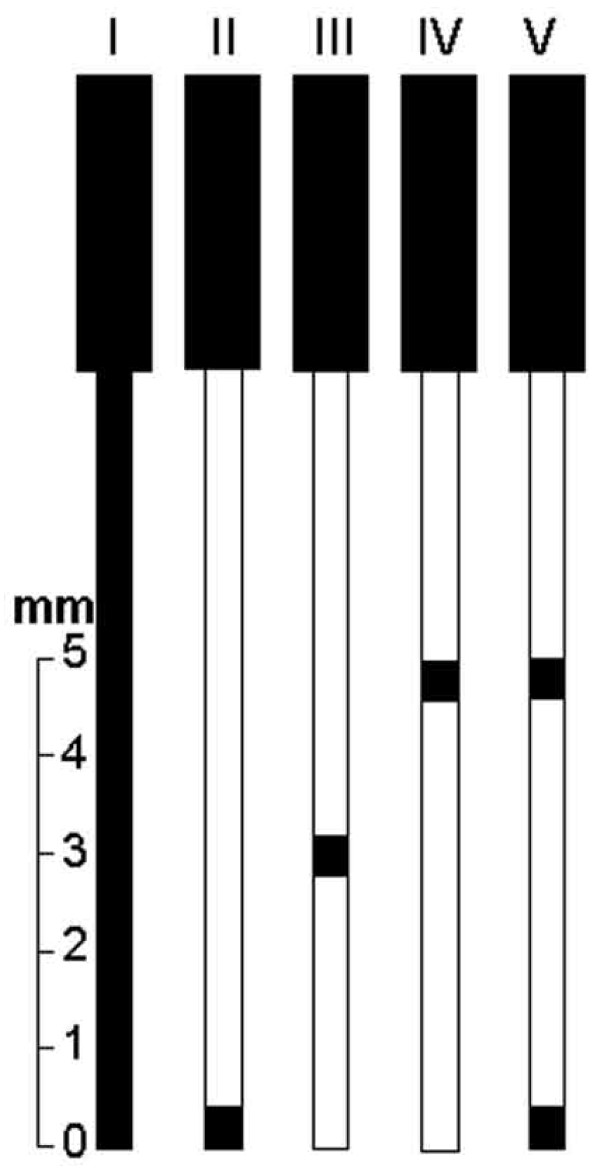
**Schematic comparison of the types of needles used in the experiment**.

The procedures were performed in the rats lightly anesthetized as described above. Uninsulated stainless steel (type I) acupuncture needles (0.18 × 8 mm, Dong Bang Acupuncture Inc., Chungnam, Korea) were used. Some needles were immersed in varnish except for the handle cable and tested with an ammeter to confirm the absence of current. These needles had the varnish circularly peeled 0.2 mm from the tip (type II), 0.2 mm at 3 mm (type III) or 5 mm (type IV) from the tip, or 0.2 mm at 5 and 0.2 mm at 1 mm from the tip (type V) as illustrated in Figure 1. The needles were inserted bilaterally at a depth of 5 mm into each hind leg at ST36 and SP6. The stimuli were generated by a constant current pulse generator model EL-608 (NKL, Brusque, SC, Brazil) and applied for 20 min to both hind legs simultaneously. The stimuli were set as alternate square waves at a frequency of 2 Hz (0.3 ms width). The current intensity was increased in a stepwise manner until a muscle twitch was observed (140-150 μA) [24,25]. The EA was performed in each rat with a current intensity of 1.4-1.5 mA that corresponded to 10 times the muscle twitch threshold [26]. Five groups of six rats each were used in these experiments. Six sham EA groups rats were placed in the same apparatus and had the type I needle insertion in the same acupoints, without the electrical current [27], examining the possibility that the simple insertion of needles caused significant nociceptive threshold influence [28].

### Injection of formalin into the acupoint

**Figure 2:**
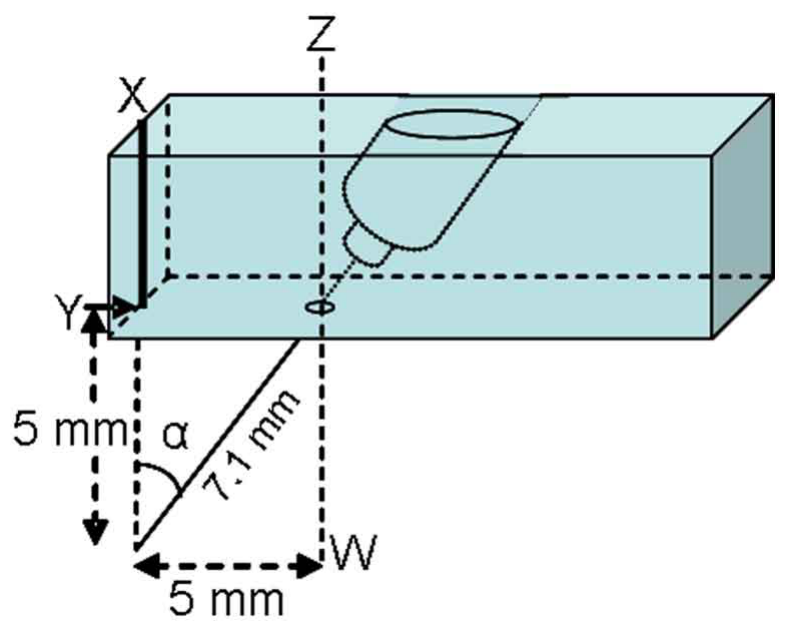
**Schematic presentation of the assembly used for the injection at an angle (α) of 45° with the line XY**. The point Y was positioned on the *Zusanli *acupoint, and the assembly was fixed by experimenter fingers against the animal leg. The line ZW, parallel to the line XY, crosses a point 5 mm distal to Y.

The injection of formalin into the acupoint was performed bilaterally in groups of six awake rats. A one-inch 25-G needle was inserted transcutaneously at 5 mm (group 1) or 1 mm (group 2) deep bilaterally into ST36 or 5 mm (group 3) deep into non-acupoints localized 5 mm posterior to ST36. In rats of group 4, the needle was inserted on a non-acupoint localized 5 mm distal to ST36. The needle was inserted at 7.1 mm deep in the cranial direction at an angle of 45° to the skin surface. A small guide built of plexiglass (Figure 2) was used to ensure that the injections in group 4 rats were made in the same position. Therefore, the needle tips in the group 1 and 4 rats were supposed to reach the same point. As a pain control, ST36 have been used for testing and evaluating EA analgesia in many experiments [29-31]. A constant volume (10 μL) of 1% formalin or saline was injected, and the syringe was then held in position for 10s and gradually removed to reduce the outflow of the drug.

### Formalin test

Each rat was injected subcutaneously with saline (10 μL) or 1% or 2.5% formalin (10 μL) into the plantar surface of a hind paw. The number of spontaneous flinching of the injected paw was counted during the 5 min of the post-injection period (first phase of the response to formalin) and then every 5 min until 60 min after the injection (second phase of the response to formalin). Flinching was characterized as a rapid and brief withdrawal or flexion of the injected paw. In separate groups of rats, the changes in the TFL measured 5 min before and 1 to 50 min after the intraplantar saline (10 μL) or 1% or 2.5% formalin (10 μL) injection were evaluated.

### Statistical analysis

The results of the changes of the formalin test were presented in graphs as the mean ± standard deviation (SD). The experimental groups were compared by multivariate analysis of variance (MANOVA) with repeated measures to compare the groups over times. The factors analyzed were treatments, time and treatment *versus *time interaction. For the significant treatment *versus *time interaction, one-way analysis of variance (ANOVA) followed by the Bonferroni correction was performed for each time. The analysis was performed using the statistical software package SPSS/PC+, version 17.0 (SPSS, Chicago, IL, USA). A probability value of *P *< 0.05 was considered to be statistically significant.

## Results

One hundred and twenty five rats were initially tested. Ninety six rats were classified as responders and were randomly assigned to 16 groups of six rats each. Twenty nine rats were classified as non-responders; thus, they were excluded from further experiments.

### The changes under 2 Hz electroacupuncture

**Figure 3:**
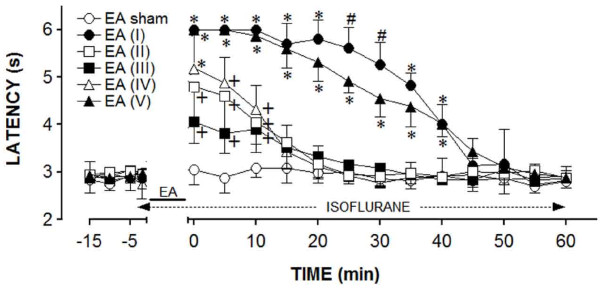
**Time course of the changes induced by the sham (EA sham) or real electroacupuncture (EA)**. Anesthesia was provided during the time indicated by a horizontal dashed arrow. EA was applied for 20 min (solid line) using the needle types numbered as in A. Points are means (± SD) of six rats per group. *P *< 0.05 compared to sham EA (*), sham EA, EA (I) and EA (V) (+), or any other group (#), using the Bonferroni *post hoc *test.

The first experiment was conducted on six groups of six "responder" rats. The control group rats were used for the EA sham, whereas the rats from the remaining groups were submitted to the EA with a particular type of needle for each group. The results of these experiments were shown in Figure 3. The rats submitted to the sham EA did not display a significant change in the TFL, as compared to the baseline TFL. In contrast, a genuine EA significantly increased the TFL, but the effect differed regarding the intensity or duration or both, which was dependent on the type of needle used. Stronger and longer-lasting effects were obtained from the rats stimulated with the type I or V needles. In these experiments, the TFL was significantly elevated, compared to the controls for up to 40 min. The EA from other types of needles increased the TFL to values significantly different from the control for up to 10 min. In addition, the effects produced by the types II, III, and IV needles were not significantly different throughout the observation period. The curves in Figure 3 were significantly different regarding the treatment (F_5,30 _= 98.04) and time (F_16,480 _= 210.76) and had a significant treatment *versus *time interaction (F_80,480 _= 24.41), *P *< 0.001 for all parameters.

### The changes of formalin injection

**Figure 4:**
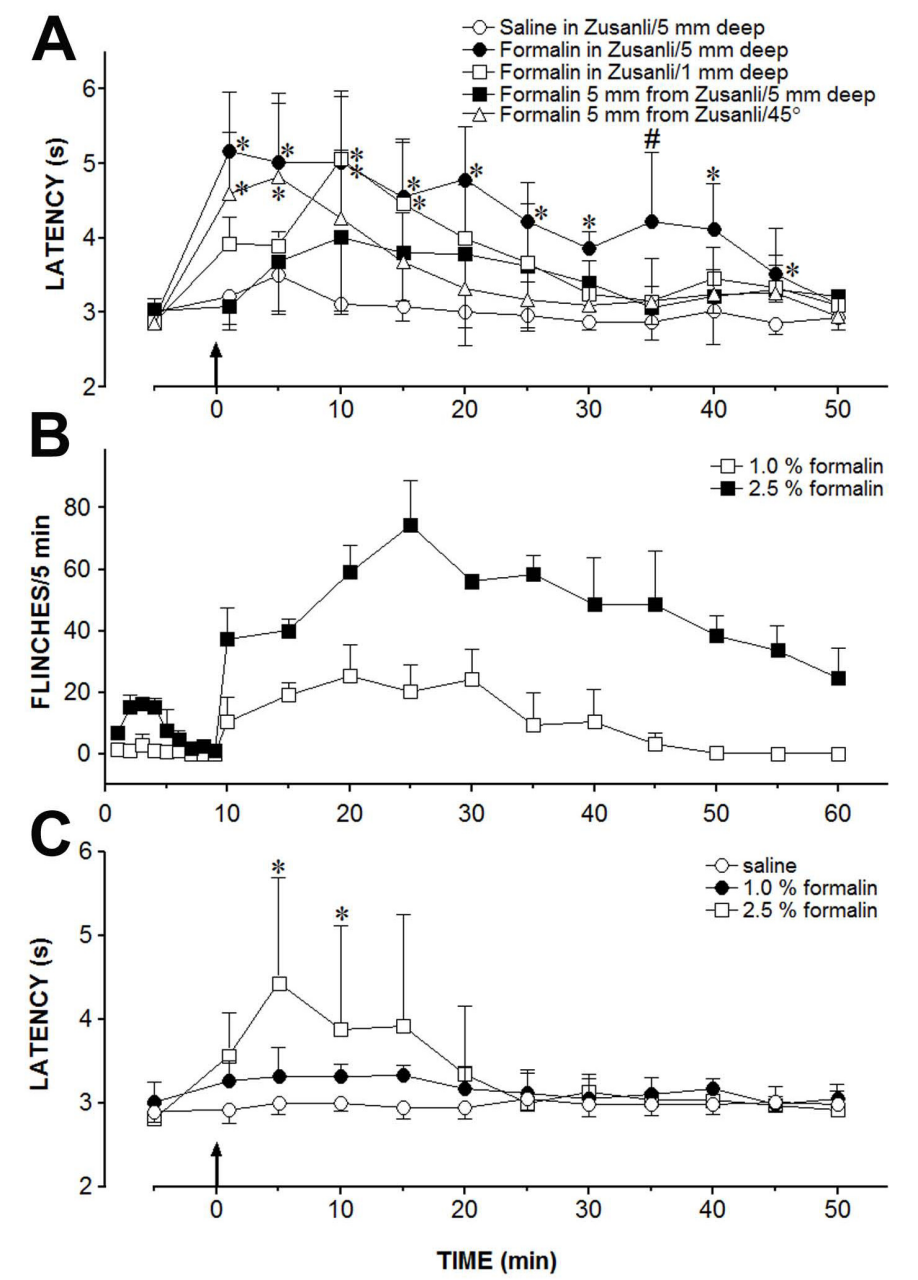
**The changes on the tail-flick latency or paw flinches produced by formalin in the rats**. (**A**) Time course of the changes induced by the injection (arrow) of saline (0.10 μL) into the *Zusanli *acupoint at 5 mm below the skin surface or 1% formalin (0.10 μL) into sites indicated in the inset on the tail-flick latency. (**B**) Time course of the changes induced by intraplantar injection of 1% or 2.5% formalin (0.10 μL) at time 0 on the cumulative flinches of the rats. (**C**) Time course of the changes induced by an intraplantar injection of saline (0.10 μL), 1% or 2.5% formalin (0.10 μL) on the tail-flick latency of the rats. Points are means (± SD) of six rats per group. *P *< 0.05 compared to saline (*) or any other group (#), using the Bonferroni *post hoc *test.

The second experiment was conducted on five groups of six "responder" rats. Saline or 1% formalin was injected bilaterally into a non-acupoint, ST36 at different depths, or 5 mm below ST36 by inserting the needle at an angle of 45° by a small guide. As shown in Figure 4A, the injection of saline (10 μL) into ST36 at a depth of 5 mm produced a non-significant change in the TFL throughout the observation period. In contrast, the injection of 1% formalin (10 μL) into ST36 at a depth of 5 mm immediately and significantly increased the TFL, compared to the control for up to 45 min. Another significant but weaker effect with a slower onset was obtained following injection of formalin into ST36 but at a depth of 1 mm. A non-significant effect was obtained following the injection of formalin into a non-acupoint 5 mm posterior to ST36 at a depth of 5 mm. An immediate and significant increase in the TFL was also obtained when formalin was injected 5 mm below ST36 by inserting the needle at an angle of 45°. However, the TFL was significantly different from the control for less than 10 min. The curves in Figure 4A were significantly different regarding the treatment (F_4,25 _= 8.72) and time (F_11,275 _= 18.11) and had a significant treatment *versus *time interaction (F_44,275 _= 2.69), *P *< 0.001 for all parameters.

### The hyperalgesic effect of intraplantar formalin

The third experiment was conducted on two groups of six rats to evaluate the classic formalin test in our laboratory conditions. Therefore, the results were not submitted to statistical analysis but were shown in Figure 4B. The subcutaneous injection of 2.5% formalin (10 μL) into the plantar surface of both hind paws produced acute nociceptive responses during the first 5 min (first phase) after the injection, and a further persistent nociceptive response was detected during a period extending from 10-60 min after the injection (second phase). A weaker (second phase) or no effect (first phase) was observed following the subcutaneous injection of 1% formalin.

### The changes of intraplantar formalin

The fourth experiment was conducted on three groups of six rats, and the results were shown in Figure 4C. The injection of saline (10 μL) into the plantar surface of both hind paws yielded no change in the TFL, whereas the intraplantar injection of 1 or 2.5% formalin (10 μL) increased the TFL. The peak effect of 2.5% formalin occurred 4 min after the injection and remained significantly above the control for another 5 min. In contrast, the effect of 1% formalin was not significantly different from the control. The curves in Figure 4C were significantly different regarding the treatment (F_2,15 _= 3.92; *P *< 0.05) and time (F_11,165 _= 4.89; *P *< 0.001) and had a significant treatment *versus *time interaction (F_22,165 _= 2.75; *P *< 0.001).

## Discussion

The efficacy of the EA stimulation depends on the distribution of the current density under the needling surface. The study demonstrated that the application of EA bilaterally to ST36 and SP6 produced antinociception in the rat tail-flick test, and the effect differed depending on the depth of the stimulation. Although the actual depth used for EA analgesia in rodents was rarely mentioned, the importance of acupuncture needle depth to produce analgesia in human has been reported [31]. Depths of 2 to 5 mm [32,33], 3 to 5 mm [34], 4 mm [35-37], or 5 mm [38-40] have been used for stimulating ST36 in the rat. A deep electrode in the EA was reported to be more effective than a surface electrode [41] because a greater number of receptors were affected [42]. We found stronger and longer-lasting (40 min post-EA) effects following the stimulation of the type I needle in the acupoints at a depth of 5 mm. Similar effects were obtained when the stimulation was applied by the type V needle. A strong but shorter effect (10 min post-EA) was obtained following the stimulation of the type IV needle. Another significant and shorter, but smaller effect (10 min post-EA) was obtained by the type II and type III needles. In the study, each needle was inserted into the acupoint so that its tip always reached a depth of 5 mm below skin surface. If the acupoint was at this depth, we should expect that the type II needle would be very effective. However, we found the EA was more effective when the type I or V needle was used. At a fixed potential, the total current delivered by an electrode increases as the electrode area increases [43]. In fact, the electric field strength is highly concentrated at the needle tip and decays rapidly with increasing distance from the tip [44], as with the type I or type V needles. Wei and Grill [43] have demonstrated that the total current increased as the electrode area increased for segmented electrode designs. We deem that the current density over the needle surface is presumed to be less intense for the type II, III, and IV needles or, instead, that the current density over the needle surface is more intense for the type V and mainly type I needles.

The duration of the effect of formalin injected into an acupoint seems to be due to volume spreading throughout the needle track. This study also demonstrated that the injection of 1% formalin (10 μL) into ST36 at a depth of 5 mm immediately and significantly increased the TFL. Chen *et al*. [18] have already demonstrated that the injection of bee venom into ST36 inhibited the persistent nociception induced by the intraplantar injection of bee venom in rats. It has also been suggested that chemical irritant acupuncture-induced analgesia was a common mechanism because similar results were also obtained with the injection of CFA or 2.5% formalin (50 μL) into ST36. We have confirmed that the intraplantar injection of 2.5% formalin evoked the classical nociceptive response, characterized by an acute first phase during a 5 min post-injection period followed by second phase characterized by prolonged nociceptive behavior for 10-60 min. The nociceptive effect of an intraplantar injection of 2.5% formalin was accompanied by a significant increase in the TFL, suggesting a diffuse noxious inhibitory control effect of formalin at this concentration. The intramuscular injection of 5% formalin or the intraplantar injection of 2% formalin produced similar noxious effects that prolonged the TFL and increased the c-Fos expression in the superficial spinal dorsal horn [45]. The intraplantar injection of 1% formalin produced a weaker (second phase) or no nociceptive effect (first phase) and did not produce any significant change in the TFL. Therefore, the antinociceptive effect produced by the injection of 1% formalin into ST36 was unlikely to result from a diffuse noxious inhibitory control effect. In present study, the peak effect of 1% formalin occurred soon after the injection into ST36 at a depth of 5 mm. However, no significant nociceptive response in rats soon after an intraplantar injection of 1% formalin was found in this study.

The injection of 1% formalin into ST36 at 1 mm from the skin surface immediately and significantly increased the TFL. However, the effect occurred 10 min later than the effect from the injection of 1% formalin into ST36 but at 5 mm from the skin surface. A non-significant and slower effect was also produced by the injection of 1% formalin 5 mm from ST36 at a depth of 5 mm from the skin surface. However, the study did not allow us to exclude that the weaker effects of the injection of formalin superficially into the acupoint or outside the acupoint were due to the drug diffusion to the acupoint at a deeper depth. Drugs spreading throughout the needle track may occur during the injection procedures despite the attempts to reduce the drug outflow. If the drug diffusion occurred, the injection into ST36 at a depth of 5 mm may be the pathway through which formalin could "wash" the entire needle track. In fact, a stronger, but much shorter, effect was obtained when formalin was injected at the same depth below ST36 when using a track beginning 5 mm caudal to the acupoint and at an angle of 45° regarding the skin surface.

Inhalation anesthesia and changes in the skin temperature are unlikely to interfere with the EA-induced antinociception in the tail-flick test. Isoflurane inhalation (1.2%) increases the tail temperature, which can obscure its antinociceptive action as evaluated by the tail-flick test [46]. A possibility still remained that our results could be influenced by the changes in the skin temperature of animals. The TFL to noxious radiant heat had been proposed to depend upon the tail temperature [47]. The changes of body temperature during EA procedures for analgesic purposes in rats have not been conducted yet. However, the skin temperatures of human volunteers decreased during the application of EA to both ST36 [48]. The systemic administration of mecamylamine, atropine or phenoxybenzamine produced significant changes in the rat tail skin temperature but none of them produced significant changes in the TFL [49]. Lichtman *et al*. [50] have also concluded that tail-skin and core temperatures have a negligible influence on the tail-flick response. In this study, the TFL obtained before and during the sham EA in rats under 0.5% isoflurane anesthesia were not significantly different. In addition, the use of isoflurane at up to 0.7% was recently shown to provide optimal conditions for the study of EA-induced analgesia in rats [51]. Therefore, inhalation anesthesia and changes in the skin temperature are unlikely to interfere with the EA-induced increase in the TFL.

## Conclusion

The experimental results suggest that the efficacy of the EA stimulation depends on the spatial distribution of the current density under the needling surface rather than only the acupoint or the depth of needling. In addition, chemical stimulation of the entire needle track below the acupoint is necessary for the acupuncture-induced antinociception in the tail-flick test.

## Abbreviations

TFL: tail flick latency; EA: Electroacupuncture; SP36: Zusanli acupoint; ANOVA: analysis of variance; MANOVA: multivariate analysis of variance; SD: standard deviation.

## Competing interests

The authors declare that they have no competing interests.

## Authors' contributions

WAP was responsible for study design, manuscript preparation and submission. MLS and JRTS were responsible for the acupuncture treatment, the behavioral tests, design and perform the statistical design and data and for manuscript review. All authors read and approved the final version of the manuscript.
